# Buffalo Breeding and Genetic Studies With a Focus on the Anatolian Buffalo, a Native Genetic Resource of Türkiye

**DOI:** 10.1002/vms3.71104

**Published:** 2026-07-22

**Authors:** Yusuf Özşensoy

**Affiliations:** ^1^ Faculty of Veterinary Medicine Department of Veterinary Genetics Sivas Cumhuriyet University Sivas Türkiye

**Keywords:** Anatolian buffalo, *Bubalus bubalis*, genetic studies, milk and meat yield, gene regions

## Abstract

Buffalo typically inhabits tropical and subtropical forests, wet grasslands with abundant rainfall or water and swampy areas. Globally, buffalo husbandry represents the second most widespread livestock activity after cattle. In many countries, buffaloes are of primary economic importance for both milk and meat production. The aim of this review is to focus on global buffalo husbandry with a specific emphasis on the Anatolian buffalo, Türkiye's native genetic resource and sole buffalo breed, while providing an overview of genetically important gene regions. The review is divided into two main sections. The first section, entitled ‘Buffalo Population and Husbandry', discusses global buffalo population numbers, country‐specific trends over the years, buffalo species and observations and recommendations regarding husbandry practices. This is followed by an analysis of buffalo population trends and related assessments in Türkiye. The second section, ’Buffalo Genetics, Genetic Studies, and Gene Regions', begins with a brief overview of general buffalo genetics and genetic diversity (characterization) studies. It then introduces gene regions that are central to buffalo production traits, specifically meat and milk, highlighting any reported significant alleles. Subsequently, genetic studies conducted on the Anatolian buffalo targeting these gene regions are summarized. To illuminate the breed's distinctive characteristics, attention has been given to the provinces from which samples were collected. For ease of comparison, the headings for gene regions in buffalo husbandry have been maintained in the section dedicated to the Anatolian buffalo.

## Graphical abstract

The Anatolian water buffalo is Türkiye's only water buffalo breed and a native genetic resource. The population of the Anatolian water buffalo declined by 76.86% between 1991 and 2010. Later, breeding programs were launched to increase the population, which reached 164,785 head by 2025. The first study on the genetic diversity of the Anatolian water buffalo was conducted in 2007. In addition, the first study focusing on specific gene regions was conducted in 2010 for PRL gene; after 2012, there was an increase in genetic studies focusing on different gene regions in Anatolian water buffalo populations from various provinces across Türkiye.

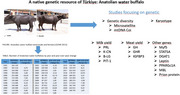

## 1 Introduction

Buffalo is the second most commonly raised species after cattle for meat and milk in many countries around the world. This species is not only important for countries' economies but also constitutes their native breeds, which are their genetic resources. In Türkiye, the only water buffalo breed raised in this context is the Anatolian water buffalo. This review focuses on buffalo breeding and genetic studies specifically related to the Anatolian water buffalo, which is Türkiye's native genetic resource. The buffalo population in the world and Türkiye and its changes over the years will be discussed. Subsequently, the description of gene regions related to productivity will be provided, and genetic studies conducted on the Anatolian water buffalo will be summarized.

## 2 Buffalo Presence and Breeding

### 2.1 Global Presence of Water Buffalo

The water buffalo (*Bubalus bubalis*), a type of livestock, has adapted to diverse climates and vegetation, including tropical and subtropical regions. It is widely raised in such countries and often lives longer than cattle (Borghese 2013a; Trapanese et al. 2024). While the species originated in Asia and is predominantly farmed there, its population is growing globally. According to various reports, the worldwide buffalo population was 168 million in 2005 (Borghese and Mazzi 2005), 182 million in 2013 (Borghese 2013a) and over 205 million by 2020 (FAO 2025). The most recent data from 2023 shows that the number has now surpassed 209 million (FAOSTAT 2025). More than 83% of the world's buffalo population is found in Southeast Asian countries, with India, Pakistan and China ranking first, second and third, respectively. When other Asian nations are included, Asia's share of the global buffalo population was 95% in 2013 (Borghese 2013a) and 97% in 2019 (Borghese et al. 2022). In Africa, domesticated buffalo are primarily found in Egypt, accounting for 2.9% of the world's population in 2013 and 1.7% in 2019. It is also worth noting the presence of wild buffalo species on the continent. In the Americas, Brazil's population represented 1.9% of the global total in 2013, which decreased to 1.23% in 2019 (Borghese 2013a; Borghese et al. 2022). The number of buffaloes in Europe has remained relatively stable. It was 500,000 in 2005 (Borghese and Mazzi 2005), dropping to about 459,000 in 2013, representing 0.2% of the global population (Borghese 2013a). This proportion remained at 0.22% (466,175 head) in 2019 (Borghese et al. 2022), in line with the overall population trend. Italy alone accounts for around 400,000 of Europe's buffalo (Borghese 2013a; Borghese et al. 2022). Despite its smaller numbers compared to Asia, Italy's buffalo industry holds a significant market position, known for high‐quality products like mozzarella cheese, fresh and processed meat and genetically superior semen (Borghese 2013a).

Buffalo are typically raised on small family farms for milk production and as working animals, especially for rice cultivation (Borghese 2013a; FAO 2025). Due to the lack of consistent animal registration systems in many countries, an accurate count of buffalo numbers is often difficult. To address this, ICAR organized a workshop and published a book on national and farm‐level strategies for buffalo population and milk production in 14 countries, including India, Bulgaria, Italy, Pakistan, Nepal, Egypt, Iran, Azerbaijan, Bangladesh, Thailand, Vietnam, Armenia, Macedonia and Iraq (Moioli et al. 2000). Statistical studies on buffalo and their products, particularly milk and meat, have also been conducted in countries such as Romania (Chetroiu and Marin 2021), rural China (Yang et al. 2007), Australia (Allen 2002) and Sri Lanka (Somapala 2002). There have also been numerous studies on buffalo welfare, especially in Italy and Brazil, though more research is needed in this area (Trapanese et al. 2024). Furthermore, studies in Nepal have investigated the genetic parameters of key economic traits in buffalo (Sah et al. 2024).

*B. bubalis*, commonly known as the water buffalo, Asian buffalo or domestic buffalo, is genetically divided into two distinct subspecies based on chromosome number: The River and Swamp buffalo (Michelizzi et al. 2010; Borghese 2013a). Both the Asian and Mediterranean buffalo are considered River buffalo. It is believed they were domesticated from the wild Indian buffalo approximately 5,1000 years ago in China and Mesopotamia. From there, they were introduced to Egypt and Italy in the 8th century via major river routes (from Indo to Tigris and Euphrates, Nilo, Sele and Volturno rivers). They were later brought to Türkiye and the Balkans in the 15th century (Borghese 2013a, 2013b).

The water buffalo is a crucial species contributing to the global agricultural economy through its meat, milk, labour and hide. Among these, meat and milk are the primary economic drivers, supporting a larger human population compared to other livestock (Xuan 2024). The river buffalo, the most numerous and economically significant type, is predominantly found in India and Pakistan. Conversely, the Swamp buffalo is widespread throughout Southeast Asia (Iannuzzi 2013; Xuan 2024).

River buffalo are physically more developed than their Swamp counterparts, and they are valued as a domestic breed due to their higher milk yield and the superior quality of their dairy products. Several River buffalo breeds have been selectively bred for milk production, including the Mediterranean Italian buffalo in Italy, the Murrah and Jafarabadi in India and the Nili‐Ravi and Kundi in Pakistan. Italy has successfully enhanced the genetic potential of its buffalo, with some individuals now producing up to 5,000 kg of milk in a 270‐day lactation period. This milk is used in a wide variety of dairy products. In addition, many countries also raise buffalo for meat production (Borghese 2013a, 2013b). Globally, buffalo milk ranks second in the dairy industry, with Italy being a top producer. To further boost milk yield and economic sustainability, research is ongoing to optimize buffalo diets with better nutrition (Infascelli et al. 2025).

While Italy focuses primarily on dairy, many other countries, including Türkiye, raise buffalo as a dual‐purpose breed for both meat and milk, where they play a crucial role in the national economy. Traditionally, buffaloes are raised in small herds and mate naturally. However, their economic importance has led to controlled breeding and selection. Consequently, countries are developing genetic strategies to improve their local buffalo breeds. For example, studies on breeding and genetic strategies have been conducted in regions like Punjab (Aulakh and Sharma 2012), Iran (Safari et al. 2018), Indonesia (Tsuji et al. 2022), Italy (Carpio, Cimmino, et al. 2023; Carpio, Cesarani, et al. 2023) and Madhya Pradesh (Shakya et al. 2023). In Southeast Asian countries, where the largest buffalo populations are found, the status of reproductive biotechnologies for enhancing production efficiency and genetic quality has also been evaluated (Hufana‐Duran et al. 2025). Given its superior genetic potential for milk production, the Italian buffalo breed has been used to create hybrid animals. Its semen has been imported by many countries, including Türkiye, other parts of Asia and even the Americas, to improve local herds (Borghese 2013b).

After milk, meat production is the next most significant economic product from River buffalo. In 2023, approximately 28.5 million buffalo were slaughtered worldwide, yielding 7.09 million tons of meat. Like milk production, Asia leads this sector, producing 96% of the world's total meat (6.84 million tons) (FAOSTAT 2025). The quantity and quality of buffalo meat is generally low because the industry primarily relies on older, retired animals. Very little meat comes from younger buffalo. To address this, it has been suggested that a dedicated meat supply chain needs to be organized to ensure a consistent and high‐quality product (Di Stasio and Brugiapaglia 2021).

### 2.2 Anatolian Water Buffalo Breed

**FIGURE 1:**
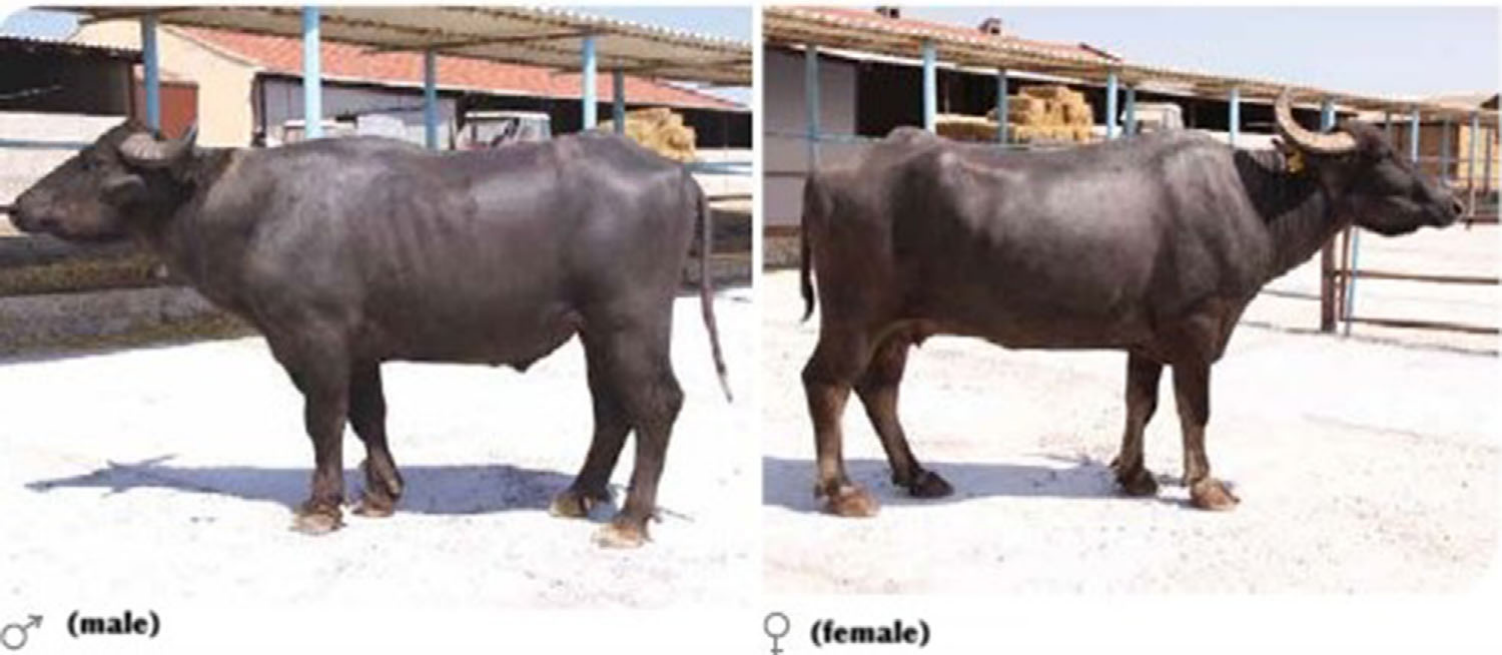
Anatolian water buffalo bred (male and female) (GDAR 2011).

All buffalo raised in Europe and the Near East are River buffalo. The species found in Türkiye is known as the Anatolian buffalo (Figure 1). This is the sole breed that has been raised in Türkiye for centuries, believed to have migrated from India. In 2004, the Anatolian buffalo was officially registered as a native breed under the Communiqué on the Registration of Local Animal Breeds and Lines (Governmental Newspaper 2004). Locally, it is also called camız, dombay, çamış and kömüş. Anatolian buffalo are spread across the country, though the majority are concentrated in the Black Sea region (Governmental Newspaper 2004). They are typically raised in small herds (83% of herds have 1–5 head, and 17% have 8 head) on a tether or in paddocks in many provinces, including Northern Central Anatolia, Thrace, Hatay, Muş, Kars, Diyarbakır, Afyonkarahisar and Sivas (Governmental Newspaper 2004; Borghese and Mazzi 2005; Borghese 2013b). To improve their genetic makeup and milk yield, Anatolian buffalo were artificially inseminated with semen imported from Italy in 2002 at the village of Ilıkpınar in Hatay (Borghese and Mazzi 2005; Borghese and Vittori 2013). The resulting F1 individuals showed an increase in average live weight and milk quantity, as well as higher milk fat and protein content (Borghese and Vittori 2013). The Anatolian buffalo is a dual‐purpose animal used for meat and labour in addition to milk. The meat yield averages 50.61% with a maximum of 54.8% (Governmental Newspaper 2004). It has also been reported that the carcass loss during slaughter is 10% less in buffalo compared to cattle (Borghese and Vittori 2013).

According to FAO data, the number of water buffalo in Türkiye was reported as one million head in 1974 (Borghese and Vittori 2013). The population of the Anatolian water buffalo declined to 366,150 in 1991 and continued to decrease annually until 2010, reaching 84,726 head. Subsequently, with the initiation of the ‘Buffalo Breeding Projects under Farmers’ Conditions', buffalo husbandry began to be promoted across the country. Despite slight declines in 2022 and 2023, the population increased overall on an annual basis and reached 164,785 head in 2025, representing a 1.69% rise compared to the previous year (Table 1) (TÜİK 2026). The ’In situ Breeding Projects' have played a significant role in this increase. In fact, it was reported that in 2024, government support for the in‐situ breeding projects of the Anatolian water buffalo would continue across 18 provinces (Governmental Newspaper 2024).

**TABLE 1:** Number of Anatolian water buffaloes by year and year‐over‐year change.

Year	Buffaloes number (Head)	Year‐over‐year change (%)	Year	Buffaloes number (Head)	Year‐over‐year change (%)
1991	366,150	**—**	2009	87,207	1.05
1992	352,410	−3.75	2010	84,726	−2.84
1993	316,000	−10.33	2011	97,632	15.23
1994	305,000	−3.48	2012	107,435	10.04
1995	255,000	−16.39	2013	117,591	9.45
1996	235,000	−7.84	2014	122,114	3.85
1997	194,000	−17.45	2015	133,766	9.54
1998	176,000	−9.28	2016	142,073	6.21
1999	165,000	−6.25	2017	161,439	13.63
2000	146,000	−11.52	2018	178,397	10.50
2001	138,000	−5.48	2019	184,192	3.25
2002	121,077	−12.26	2020	192,489	4.50
2003	113,356	−6.38	2021	185,574	−3.59
2004	103,900	−8.34	2022	171,835	−7.40
2005	104,965	1.03	2023	161,749	−5.87
2006	100,516	−4.24	2024	162,051	0.19
2007	84,705	−15.73	2025	164,785	1.69
2008	86,297	1.88			

In Türkiye, the amount of milk obtained from water buffalo was 43,025 tons in 2023, while in 2024 this quantity increased by 35.1%, reaching 58,122 tons. The share of buffalo milk in total milk production was 0.2% in 2023, rising to 0.3% in 2024 (TÜİK 2025a). Globally, buffalo milk ranks second in the dairy industry in terms of total milk production; however, in Türkiye, its share in overall milk production remains relatively low. Türkiye's total red meat production decreased by 11.7% from 2023 to 2024. Within this context, buffalo meat production fell from 15,386 tons in 2023 to 13,781 tons in 2024, corresponding to a 10.4% decline. Despite this reduction, the proportion of buffalo meat in total red meat production rose from 0.6% in 2023 to 0.7% in 2024 (TÜİK 2025b). The horns of Anatolian water buffalo have traditionally been used in the making of Turkish bows and ney mouthpieces, while their meat is utilized in sausage production. Their milk is processed into clotted cream (kaymak) and mozzarella cheese, and the most widely produced dairy products include various cheeses and ayran made from buffalo yogurt (GDAR 2011; Borghese 2013b). Furthermore, in the traditional cuisine of Afyonkarahisar—a province with an important place in Türkiye's gastronomy—buffalo milk, kaymak and yogurt are frequently consumed, with kaymak being the most preferred product. However, it has been reported that the decline in buffalo numbers has negatively affected the availability and use of buffalo‐derived products (Özer et al. 2022).

#### 2.2.1 Limitations of Buffalo Farming

The importance of the buffalo has been explained above. However, there are also significant problems. The most significant problem in buffalo farming is the difficulty of managing herds due to the animals’ physical characteristics. In addition, since farming is based on profit generation, when breeding a particular breed, the farmer focuses on that breed's operational benefits and income potential. In this context, the availability of markets for buffalo products and market share are seen as significant problems in regions where buffalo are raised. Compared to cattle bred for high milk and meat yields—key products—buffalo have lower milk and meat yields, which is their most significant disadvantage. However, buffalo meat is not a widely preferred product in Türkiye.

A study was conducted in Türkiye with the aim of raising awareness regarding the status of water buffalo husbandry, focusing not only on the population dynamics but also on the decline in buffalo‐derived products such as meat, milk and hides. This research evaluated 38 years of data (1970–2008) (Sarıözkan 2011). In addition, another study utilized data on water buffalo in Türkiye from 1991 to 2022 and, through the application of the R programming language, attempted to forecast the state of buffalo husbandry between 2023 and 2032. According to this projection, the buffalo population is expected to decline annually, reaching as few as 19,136 head by 2032. The underlying reasons for this reduction were reported to include the preference for higher‐yielding cattle breeds, increasing mechanization, the shrinking of suitable habitats for buffalo breeding and rising input costs (M. Özdemir et al. 2024). Furthermore, research has been conducted to identify the key agricultural policy components that should be prioritized in order to develop optimal strategies for buffalo breeding in Türkiye, with the goal of ensuring the welfare of buffalo farmers and enhancing production efficiency (Taşcioğlu et al. 2020). In Iğdır province, investigations focused on milk production and utilization among buffalo breeders. Findings emphasized not only the use of systematic breeding methods but also the necessity of establishing cooperatives for marketing purposes (Yılmaz et al. 2023).

Farmers demand greater quantity and quality of products derived from buffalo meat and milk. Consequently, as technology becomes increasingly integrated across all sectors, agriculture has also begun adopting advanced methods to achieve economic, environmentally sustainable and high‐quality production. In Yozgat province, a SWOT analysis was carried out with buffalo breeders to evaluate the applicability of Precision Livestock Farming (PLF) practices. The study demonstrated that the use of PLF technologies could enhance both the quantity and quality of buffalo meat and milk in a controlled manner, while simultaneously reducing production costs (Ermetin 2023).

In a survey conducted with water buffalo farmers in the province of Bingöl, Türkiye. The farmers reported that their most significant challenges were high feeding costs and the absence of grants and loan support, and they emphasized the need for improved marketing opportunities for their products. In addition, they highlighted the need to improving genetic quality of the animals (G. Özdemir and Özdemir 2016). In addition to these, various studies have investigated and assessed the current status, challenges and potential solutions for buffalo husbandry and production in Türkiye, including in provinces with the highest buffalo populations (Gül et al. 2018) as well as in several other regions (Tekerli and Gündoğan 2005; Aygün 2021; Turan and Tatar 2022).

## 3 Buffalo Genetics and Research

### 3.1 Overview

Buffalo are generally classified into two species: The African buffalo (*Syncerus caffer*) and the Asian buffalo (*B. bubalis*). Each of these species comprises two subspecies. The African buffalo inhabits the wild in countries such as Ethiopia, Somalia, South Africa and Kenya. Among its subspecies, the Savannah or Cape buffalo (*S. caffer caffer*) possesses 52 chromosomes, whereas the forest buffalo (*S. caffer nanus*) carries 54 chromosomes. The subspecies of the Asian buffalo include the river buffalo (*B. bubalis bubalis)*, which has 50 chromosomes, and the swamp buffalo (*B. bubalis carabanesis)*, which has 48 chromosomes. Hybridization between subspecies of both species can yield viable and fertile offspring with 53 and 49 chromosomes, respectively (Michelizzi et al. 2010; Iannuzzi 2013).

The first complete mitochondrial DNA (mtDNA) sequencing study in buffalo was accomplished in Indian water buffalo in 2002 by Verma et al. (unpublished), generating a sequence of 16,358 base pairs (https://www.ncbi.nlm.nih.gov/nuccore/AF547270). In swamp buffalo, this was completed in 2004 by Qian et al. (unpublished) (Pariset and Valentini 2013). The reference genome assembly of the buffalo was fully achieved in 2019, utilizing single‐molecule sequencing and chromatin conformation capture data to generate a chromosome‐level genome assembly (Low et al. 2019).

According to the OMIA (Online Mendelian Inheritance in Animals) database, in buffalo there are 44 traits in total (both disease‐related and non‐disease‐related); nine monogenic traits with at least one known variant and five monogenic traits for which all variants have been identified (disease‐related or not). In addition, three monogenic diseases with at least one known variant and two monogenic diseases with all variants identified have been reported. Among these, 22 traits have been described as potential models for human diseases (Omia 2025).

### 3.2 Studies on Buffalo Genetics and Gene Sites

Molecular genetic studies in buffalo have generally been considered to have started relatively late compared to other domestic species. The research conducted to date has primarily focused on comparative analyses of river and swamp buffalo with respect to mtDNA, microsatellites, protein loci and Y‐chromosome genes (Pariset and Valentini 2013). Following the establishment of the buffalo reference genome sequence (Low et al. 2019), genetic studies in buffalo gained momentum and omics approaches began to be employed to assist in the selection of animals, alongside genomic prediction, genome‐wide association studies (GWAS), evolutionary biology and functional genomics (Abdel‐Shafy et al. 2022).

In Italy, which has the largest buffalo population in Europe and plays a leading role in milk production, a genomic selection study was conducted in Italian Mediterranean buffalo, bred for milk and mozzarella production, to assess the feasibility of applying genomic selection (Cesarani et al. 2021). In recent years, genetic research in buffalo has accelerated, and using contemporary technologies such as cloning, numerous clones have been produced from superior bulls in India—the first in 2009 and the second in 2020. Multiple parameters have been evaluated in these clones (Yadav et al. 2020, 2024).

Following these general genetic studies and the discussion of information regarding buffalo genetic characterization, the review focus will shift to genomic regions associated with production traits, including milk yield, meat production and live weight in buffalo.

#### 3.2.1 Genetic Diversity

##### 3.2.1.1 General Information About Buffaloes

Determining genetic diversity both within and between buffalo breeds plays a crucial role not only in the management and breeding programs of the breed but also in its conservation (Xuan 2024). To this end, a wide range of molecular approaches are employed. Molecular markers are used in buffalo for purposes such as phylogenetic analyses, single nucleotide polymorphism (SNP) discovery and characterization, population breeding and drift studies, marker‐assisted selection (MAS), genetic improvement strategies and genomic selection within breeding programs (Xuan 2024).

The initial genetic studies in buffalo focused on investigating genetic variation within Asian buffalo populations, particularly examining variations in protein‐coding loci and microsatellite loci to assess genetic diversity (Barker, Tan, et al. 1997; Barker, Moore, et al. 1997). Using microsatellite markers, genetic diversity studies conducted on Iranian buffalo (Darestani et al. 2019) and Indonesian swamp buffalo (Gunawan et al. 2022) demonstrated high levels of genetic variation both within and between populations. Another study on genetic diversity in buffalo employed the mtDNA cytochrome b region to analyse two buffalo types (river and swamp) in the Philippines, confirming once again the clear differentiation between these two subspecies (Cailipan et al. 2023).

##### 3.2.1.2 About Anatolian Water Buffalo Breed

In the first study aimed at determining the genetic diversity of Anatolian buffalo, cattle microsatellites were analysed in buffalo located in Istanbul, and 4 out of 11 markers were found to be polymorphic in buffalo (Soysal et al. 2007). Subsequently, a genetic diversity study was conducted on buffalo populations from 17 provinces across six different geographical regions of Türkiye using 20 buffalo‐specific microsatellite markers. This study revealed high genetic diversity and polymorphism within two major clusters, with the observed differences primarily attributable to individual variation. Notably, buffalo from the Black Sea region formed a distinct cluster among themselves (Ünal et al. 2021).

In the most recent study, molecular identification, and taxonomic classification of 20 male Anatolian buffalo from the Tokat region were carried out using the mtDNA COI gene region for barcoding. The samples were compared against the databases of *B. bubalis*, *B. carabanensis* and *Bos taurus*, confirming that the Anatolian buffalo clustered within the *B. bubalis* group (Güllüce and Şahin 2025).

Karyotype studies have also been conducted to determine the chromosome number and characteristics of Anatolian buffalo. Although Anatolian buffalo are reported to belong to the river type, no prior karyotype analysis had been performed. In the first karyotype study, five healthy male Anatolian buffalo at the Balıkesir Marmara Research Center were analysed, revealing that they possess 50 chromosomes, the same number as river buffalo (Yavaşoğlu et al. 2014). In another study conducted on Anatolian buffalo in Tekirdağ, it was confirmed once again that these buffalo have 50 chromosomes, and the GTG banding method was applied for the first time (Soysal et al. 2016).

#### 3.2.2 Genes Related to Milk Yield

##### 3.2.2.1 General Information About Buffaloes

Prolactin (PRL) is a polypeptide hormone synthesized and secreted by specialized cells of the anterior pituitary gland, and it is also present in the mammary epithelial cells during lactation as well as in the milk itself. This hormone plays a critical role not only in the growth and development of mammary glands and secretory tissues but also in key biological processes such as reproduction and homeostasis. Given that PRL has a central regulatory role in mammary gland development, milk secretion and the expression of milk protein genes, it is considered an important genetic marker for production traits in dairy animals. Notably, its third exon contains two major allelic variants (B and b) that are associated with milk yield (Freeman et al. 2000; Alipanah et al. 2007).

In ruminants, approximately 95% of milk proteins are composed of alpha‐lactalbumin, beta‐lactoglobulin (B‐LG) and four caseins (αs1‐, αs2‐, β‐ and κ‐casein), with caseins alone accounting for nearly 80% of the total protein content (Gangaraj et al. 2008; Ramesha et al. 2016). The most common variants of β‐casein are A1 and A2. The presence of the A1 variant in milk has been associated with health concerns and is therefore considered undesirable. In buffalo milk, the A2 variant of these proteins has been detected at a high frequency (Ramesha et al. 2016).

Kappa‐casein (K‐CN) constitutes approximately 12% of total casein proteins. Among the six alleles of this gene, the most common are the A allele, which is associated with high milk yield but lower milk protein content, and the B allele, which is linked to higher milk fat and protein content, resulting in superior milk quality but lower overall milk yield. The BB genotype has been reported to contribute significantly to genetic variation related to high protein content (Gangaraj et al. 2008). Because the B allele is more prevalent, it is recommended that animals carrying this allele be preferentially used both in dairy operations and artificial insemination programs (Mitra et al. 1998; Abdel Dayem et al. 2009). In addition, the E allele has been reported to negatively affect milk protein quality and is therefore considered undesirable in the herd (Matějíček et al. 2007).

In a recent study, associations between SNPs and milk yield were investigated in swamp buffalo. In this study, 40 SNPs were genotyped, and a total of 10 SNPs were found to exhibit polymorphisms associated with milk production. These included two SNPs related to hormones (both in the leptin gene), six SNPs related to energy balance (two in GHRL, one in ARHGAP39 and three in MC4R), and two SNPs associated with the immune system (one in BOLA‐DQA1 and one in TFAP2D) (Boonyanuwat et al. 2025).

##### 3.2.2.2 About Anatolian Water Buffalo Breed

The PRL gene was investigated in 45 samples collected from four provinces (Amasya, Afyonkarahisar, Konya and Sivas) of Anatolian buffalo, and it was determined that the exon 3 region of the gene was monomorphic, with all samples exhibiting the AA genotype (Kaplan and Boztepe 2010). In another study on Anatolian buffalo raised in four other provinces (Kayseri, Afyonkarahisar, Amasya and Çorum), the AB genotype was detected in 81% of the samples, and the A allele frequency was 55% (Konca and Akyüz 2017). In a further study examining both exons 1 and 3 of the PRL gene using a large sample of 129 Anatolian buffalo from Sivas, the AA genotype (Hae III) and BB genotype (Rsa I) were observed in exon 1, while the AA genotype (Rsa I) was detected in exon 3 (Özşensoy 2018). Another study on Anatolian buffalo in the Kızılırmak Delta also reported the gene to be monomorphic (Toparslan and Mercan 2019). Collectively, these results indicate that the PRL gene in Anatolian buffalo predominantly carries the A allele.

In Anatolian buffalo, a study on the K‐CN gene using samples from five provinces representing different geographic regions (Afyonkarahisar, Kayseri, Amasya, Çorum and Diyarbakır) reported only the BB genotype, whereas a study on the B‐LG gene detected the AA, AB and BB genotypes (Cınar et al. 2016). In a study conducted on Anatolian buffalo raised in Sivas, different restriction enzymes were used to identify the A, B and E alleles of the K‐CN gene. Across various regions of the gene, the AA, BB and AB genotypes were detected, while the undesirable E allele was not observed in any sample (Özşensoy 2020). Another study on Anatolian buffalo from the Kızılırmak Delta also reported the gene to be monomorphic (Toparslan and Mercan 2019). At the Sheep Research Institute in Bandırma, Balıkesir, a study involving Anatolian buffalo, Murrah buffalo and their hybrids investigated the effect of K‐CN genotypes on milk production traits. Among the three genotypes, animals with the BB genotype exhibited significantly higher lactation milk yield, suggesting that K‐CN can serve as a genetic marker in dairy buffalo breeding (Karadağ et al. 2023). Collectively, these studies indicate that the B allele, which is important for milk production, is consistently present in Anatolian buffalo. Therefore, it can be concluded that Anatolian buffalo have the potential for productive milk yield.

In a study investigating three milk production‐related genotypes—PRL, K‐CN and pituitary transcription factor 1 (PIT1)—in Anatolian buffalo from the Kızılırmak Delta, which encompasses provinces in the Black Sea region, the primary area of Anatolian buffalo husbandry in Türkiye, all genes were found to exhibit homozygous structures. Consequently, it was reported that a genetic bottleneck may have occurred in the Anatolian buffalo population within this delta (Toparslan and Mercan 2019).

A recent study examined the relationship between different milk compositions (milk fat, protein, dry matter and pH) and the expression levels of miRNA‐15a, miRNA‐29b, miRNA‐34a and miRNA‐223 in Anatolian buffalo, cow, sheep, goat and donkey milk. The study found statistically significant correlations between different compositions in other species. However, although a correlation was found between miRNA‐15a and dry matter and fat content in buffalo milk, it was not statistically significant (Celik et al. 2025).

#### 3.2.3 Genes Related to Growth and Meat Yield

##### 3.2.3.1 General Information About Buffaloes

Several hormones play important roles in meat production. One of these hormones, growth hormone (GH), is an anabolic hormone synthesized and secreted by the somatotroph cells of the anterior pituitary gland, with its release regulated by the hypothalamus. GH binds to growth hormone receptors (GHR) on the surface of target cells, exerting significant effects on growth and metabolism, with a single GH molecule binding to two GH receptors. In addition to its critical role in growth, GH is responsible for modulating protein, fat and carbohydrate metabolism (Di Stasio et al. 2005; Ayuk and Sheppard 2006). A polymorphism arises from an amino acid substitution (leucine to valine) at the 127th polypeptide position in exon 5 of the GH gene, which can be detected using the Alu I restriction enzyme. It has been reported that animals with higher birth weights often carry the LL genotype of the GH gene (Mitra et al. 1995; Biswas et al. 2003).

The GHR is a transmembrane protein belonging to the class I cytokine receptor superfamily, which binds GH upon expression (Flores‐Morales et al. 2006; Andreas et al. 2010). To identify candidate genes for meat production traits, the exon 10 region of the GHR gene has been recognized as a candidate, with the A allele associated with higher meat production‐related characteristics (Di Stasio et al. 2005).

Although six different types of the insulin‐like growth factor binding protein (IGFBP) gene (IGFBP 1–6) have been identified, IGFBP‐3 is the most abundant in the serum of both humans and animals (Shimasaki and Ling 1991). Research on the IGFBP‐3 region generally focuses on segments encompassing exon 3, the entirety of intron 2 and parts of exon 2 and intron 3, which are investigated using various restriction enzymes. Studies in buffalo have predominantly identified this region as monomorphic (Padma et al. 2004; Othman et al. 2018; Saleh et al. 2019). It has also been reported that the IGFBP‐3 region can serve as a marker for species identification (Padma et al. 2004).

##### 3.2.3.2 About Anatolian Water Buffalo Breed

In a study conducted on Anatolian buffalo raised in Sivas, two genotypes (LL and LV) were identified in the exon 4–exon 5 region of the GH gene. The LL genotype was observed at the highest frequency of 78.44%, and the L allele at 89%. In the same study, two genotypes (AA and AG) were detected in the GHR gene, with the AG genotype being the most frequent at 92.19% and the A allele at 54% (Özşensoy and Kara 2019). In another study on Anatolian buffalo from four provinces in Türkiye (Kayseri, Afyonkarahisar, Amasya and Çorum), polymorphisms in the GH gene region were observed as LL (75.5%), VV (0.017%) and LV (22.8%), with an L allele frequency of 87%. In this study, only the AA genotype was detected in the GHR gene (Konca and Akyüz 2017). These results indicate that the LL genotype in the GH gene and the A allele in the GHR gene are prevalent in relation to meat production, suggesting that Anatolian buffalo are an important breed for meat yield.

In a study analysing the sequence of the GH gene using samples collected from 17 provinces, four genotypes (with eight SNPs) were identified in the same gene region, and seven genotypes (including 20 SNPs and one del/ins) were identified in the exon 3–intron 3–exon 4 region. These newly identified polymorphisms are reported to potentially affect economically important traits in buffalo, including body composition, carcass characteristics, reproduction and milk yield and composition (Ünal et al. 2020). In addition, in Anatolian buffalo from Afyonkarahisar, SNPs in the GHR gene were investigated for associations with growth and milk yield. One mutation (g.31601784 G>A) was found to influence growth in animals younger than one year, while another mutation (GG genotype at g.31601783) was associated with higher milk production (Erdoğan et al. 2021).

In a study conducted on Anatolian buffalo in Sivas, the IGFBP‐3 gene was investigated for the first time. Different regions of the gene were analysed, and in addition to previously reported alleles, three novel genotypes—located in the exon 2–intron 3 and intron 2 regions—that had not been identified in any other buffalo breed were detected for the first time in Anatolian buffalo. It was reported that one of these newly identified alleles exhibited a very high frequency and that these novel alleles and genotypes might be unique to Anatolian buffalo (Özşensoy and Baral 2023). While this gene region has been found to be monomorphic in buffalo populations from other countries, it was polymorphic in this study. The identification of these novel genotypes for the first time is considered evidence that Anatolian buffalo represents a breed with distinct genetic characteristics.

#### 3.2.4 Other Genes

##### 3.2.4.1 General Information About Buffaloes

The four genes belonging to the myogenic regulatory factor (MRF) family—Myf5, MyoD, Myogenin and Myogenic regulatory factor 4 (MRF4)—are expressed in these cells during embryogenesis and postnatal myogenesis. They have been shown to control the determination and differentiation of skeletal muscle cells and to be functionally interrelated. Following the discovery of these genes, significant progress was made in understanding the regulation of myogenic differentiation, and their roles in skeletal muscle regeneration were elucidated. Notably, the absence of Myf5 and MyoD has been reported to severely disrupt multiple stages of the myogenic program (Hernández‐Hernández et al. 2017; Zammit 2017).

Signal transducers and activators of transcription (STAT) proteins constitute a family of latent cytoplasmic proteins that become activated in response to various extracellular polypeptides, enabling them to participate in gene regulation. Numerous genetic studies have identified these proteins as transcription factors that play critical roles in cytokine‐mediated signalling pathways. Moreover, STAT activation has been shown to be of significant importance in growth regulation in animals, as well as in the normal progression of the cell cycle and the regulation of apoptosis (Bromberg and Darnell 2000).

Triacylglycerols (TAGs) are the primary constituents of intramuscular fat (IMF) and milk fat. IMF is also associated with meat production in animals. Therefore, understanding the polymorphisms and genes related to fat synthesis is of significant importance in livestock production. Acyl‐CoA: diacylglycerol acyltransferase 1 (DGAT1) encodes a microsomal enzyme, diacylglycerol O‐acyltransferase, which catalyses the final step of triglyceride synthesis. Given its critical role in fat metabolism and TAG synthesis, DGAT1 plays an important role in both milk and meat production in animals. Associations between DGAT1 and production traits, as well as specific polymorphisms, have been reported in cattle, buffalo, goats and sheep. The absence of either allele of this gene has been shown to result in insufficient triglyceride synthesis in the mammary gland, leading to an inability to secrete milk, thereby establishing DGAT1 as a functional candidate gene for milk production. In particular, the lysine‐232 to alanine mutation in the DGAT1 gene has been associated with milk fat content in cattle (Winter et al. 2002; Khan et al. 2021).

Leptin has been found to be expressed in adipose tissue in mammals. Circulating leptin concentrations have been shown to correlate closely with the mass of adipose tissue, indicating that leptin serves as an excellent biomarker for body fat content. Although leptin is also expressed in tissues other than adipose tissue, the primary source of circulating leptin is adipose tissue. Plasma leptin concentrations vary according to factors such as body fat mass, sex, fat distribution and nutritional status, and leptin has been reported to play a significant role in reproductive performance (Zhang and Chua 2018).

Peroxisome proliferator‐activated receptors (PPARs) regulate various cellular functions primarily by controlling genes related to tissue development, bioenergetics and inflammation. PPARs were first identified in 1990 with the alpha subtype, which is activated by peroxisome proliferators at micromolar concentrations, followed by the identification of the beta and gamma subtypes in 1992. Although these three subtypes are interrelated, each is distinct and expressed in a tissue‐specific manner. The alpha and beta subtypes are highly expressed in a few tissues, whereas the gamma subtype is expressed in multiple metabolically active tissues. PPAR gamma plays a central role in insulin sensitivity and lipid metabolism by regulating genes involved in lipid uptake, synthesis and storage (Kliewer et al. 2001; Bervejillo and Ferreira 2019). The peroxisome proliferator‐activated receptor gamma coactivator 1 Alpha (PPARGC1A) gene plays a key role in energy, fat and glucose metabolism. In cattle, it has been reported that different genotypes of this gene are associated with milk fat yield, and a SNP located in intron 9 has been suggested as a candidate marker for this trait (Weikard et al. 2005).

In a recent study, transcriptomic maps at the cellular level were generated for river and swamp buffalo, both of which are Asian buffalo, with the aim of elucidating the differences in milk production at the level of cell types and gene expression (Dai et al. 2025).

##### 3.2.4.2 About Anatolian Water Buffalo Breed

In a study on Anatolian buffalo from Kütahya and Konya, variants of the Myf5 gene were investigated using seven different restriction enzymes. Six of these enzymes were found to be monomorphic, whereas one enzyme (BsuRI) revealed polymorphism, and a novel SNP was identified at the C14553A position of the gene (Bayraktar 2022). In a study conducted on Anatolian buffalo from Afyonkarahisar, polymorphisms in exons 6–9 of the STAT5A gene were examined, and only a single polymorphism (a silent mutation) was detected in exon 8 (İbiş and Erdoğan 2019). In another study examining Myf5 and STAT5A, both of which are considered candidate genes for meat and milk production in buffalo, the STAT5A gene was found to be monomorphic, while the Myf5 gene was polymorphic with three distinct genotypes (Daldaban et al. 2020).

In a study investigating nucleotide variation in exon 8 of the DGAT1 gene in 41 samples collected from Afyonkarahisar, Konya and Sivas, a total of four haplotypes were identified within two main haplotype groups (the Sivas group and the Konya–Afyonkarahisar group). The K allele, which has been reported to be associated with high milk fat content, was detected in the buffalo analysed (Özdil and İlhan 2012). In the first study on the leptin gene in buffalo, targeting the intron 1–exon 2–intron 2 region, three genotypes (TT, TG and GG) were identified at the T1131G mutation site in 70 samples collected from Istanbul (Kaplan 2018a). In another study involving sequence analysis of the same gene region with 54 samples, seven polymorphic and one monomorphic regions were detected (Kaplan 2018b). In a study examining genetic variation in the DGAT1 and leptin genes related to reproduction, growth, milk yield and milk composition, samples were collected from 16 provinces covering five regions. This study identified 38 nucleotide variations and one indel (reported for the first time) in the leptin gene. In the DGAT1 gene, four nucleotide variations were detected. It was concluded that these mutations could potentially influence reproduction, growth and milk yield and composition in buffalo (Işık et al. 2022).

In a study on Anatolian buffalo from Afyonkarahisar, the exon 8 region of the PPARGC1A gene was analysed using sequencing, revealing a c.1598 A>T polymorphism that results in an amino acid change. The frequency of the A allele was determined to be 0.768 (Alyörük and Erdoğan 2018). In Anatolian buffalo from Konya and Kütahya, another gene investigated was the bovine smoothened (SMO) gene. A SNP (G>C) located in exon 9 of this gene was analysed and found to be monomorphic with the GG genotype in Anatolian buffalo. It was reported that this mutation is absent and that the gene is not a candidate gene for buffalo (Aytekin and Bayraktar 2021).

In the most recent study on Anatolian buffalo, multiple body measurements were taken, and a single‐step genome‐wide association study (ssGWAS) was conducted to identify genomic regions associated with body size and ultrasonographic carcass traits. The study utilized the 90K Axiom Buffalo Genotyping Array, and 20 SNPs were found to be significantly associated at the genome‐wide level. Some of these SNPs corresponded to previously identified genes known to be related to body size and fat‐related traits, while several newly identified SNPs were also detected. The identification of these genes is reported to have the potential to enhance genetic improvement and contribute to understanding the genetic basis of buffalo morphology (Çinkaya et al. 2025).

In Anatolian buffalo, which constitute a native genetic resource of Türkiye, studies have been conducted not only on gene regions associated with production traits but also on certain gene regions considered essential for effective breeding and management;

One of these studies focused on the MBL (mannose‐binding lectin) gene, which is associated with disease resistance. Anatolian buffalo from Amasya, Çorum, Kayseri and Afyonkarahisar were analysed, and polymorphism was observed in three SNPs. However, the genotype reported to confer resistance to mastitis was found at a low frequency in these buffalo (Aksel and Akyüz 2023). Another significant line of research has investigated the prion protein gene. In particular, insertion–deletion (indel) polymorphisms located in the promoter (for 23 bp) and intron 1 (for 12 bp) regions of this gene are reported to be associated with disease susceptibility. Specifically, deletion mutations in these regions confer susceptibility, whereas insertion mutations are associated with resistance. Studies on Anatolian buffalo from Afyonkarahisar, Amasya, İstanbul, Edirne and Tokat revealed a very high frequency of the insertion mutation (in12/in23 frequency: 0.86) and a very low frequency of the deletion mutation (del12/del23 frequency: 0.05) (Oztabak et al. 2009). Another study on Anatolian buffalo at the Balıkesir Sheep Research Center reported no deletions in either region, with only the insertion mutation being present (Yaman et al. 2017). Overall, these results indicate that, unlike cattle, the resistant genotype is highly prevalent in buffalo. Furthermore, since these animals have not been observed to develop the disease, the precise functional effect of these genotypes remains unclear. Consequently, the coding region of the prion protein gene was also investigated, revealing three synonymous and one nonsynonymous SNPs (Yaman and Ün 2018).

## 4 Conclusion

Throughout this review, the focus has been on the Anatolian buffalo, a native genetic resource of Türkiye, while also providing a general perspective on buffalo husbandry both globally and within Türkiye. Following a significant decline in population, the implementation of the ‘In Situ Improvement Projects’ has led to a modest increase in the number of Anatolian buffalo, although the current population remains insufficient. Without more robust interventions, however, the buffalo population is at risk of serious decline. Globally, genetic studies in buffalo have targeted specific gene regions, and while research in Türkiye is comparatively limited, diverse genetic investigations have been conducted. These studies have revealed alleles in the Anatolian buffalo that are significant for both meat and milk production, and some alleles appear to be unique to this breed. Given that the Anatolian buffalo represents a native genetic resource of Türkiye, it is imperative to implement more comprehensive conservation strategies and to expand genetic research on this breed.

## Author Contributions

**Yusuf Özşensoy**; conceptualization, investigation, methodology, data curation, writing – original draft preparation, writing – review and editing.

## Funding

The author has nothing to report.

## Ethics Statement

The authors have nothing to report.

## Conflicts of Interest

The author declares no conflicts of interest.

## Data Availability

No datasets were generated or analysed during the current study.
